# BEING BOLD: USING PUBLIC HEALTH AWARDS TO CHANGE STATE DEMENTIA POLICY

**DOI:** 10.1093/geroni/igae098.2208

**Published:** 2024-12-31

**Authors:** Annie Rhodes, Ashley Staton, Daniel Bluestein, Kimberly Ivey, Rachel Coney, Andrea Price, Lana Sargent, Melicent Miller

**Affiliations:** Virginia Commonwealth University, Richmond, Virginia, United States; Virginia Commonwealth University, Richmond, Virginia, United States; Virginia Commonwealth University, Richmond, Virginia, United States; Virginia Commonwealth University, Richmond, Virginia, United States; Virginia Department of Health, Richmond, Virginia, United States; Virginia Center on Aging, Richmond, Virginia, United States; Virginia Commonwealth University, Richmond, Virginia, United States; Virginia Department of Health, Richmond, Virginia, United States

## Abstract

The BOLD Act (Building Our Largest Dementia Infrastructure) was passed by the United States Congress on December 31st, 2018, to support state health departments with dementia-friendly and healthy brain activities. In Virginia, the Virginia Department of Health (VDH) awarded the Virginia Center on Aging a BOLD sub-award to create an epidemiologic registry to catalog dementia, neurodegenerative disorders, and caregiving cases. Registries are a valuable but underused strategy for an equitable public health response to brain health promotion and to monitor trends in dementia and neurodegenerative disorders. The Virginia registry, known as the “Virginia Memory Project” (VMP), is only the 4th statewide dementia registry in the United States. In 2024, the Virginia State legislature unanimously voted to pass HB 1455, establishing the VMP into state law and codifying a dementia and caregiving registry in Virginia. A key outcome and sustainability strategy of launching the VMP was creating a framework to influence policy at the state level to infuse public policy with evidence-based resources, education, and impact for dementia, caregiving, and neurodegenerative disorders. The VMP is unique among epidemiologic dementia registries because it is a responsive registry that offers patients, caregivers, and persons with undiagnosed memory concerns linkage to information, services, and support. In this session, we will discuss the framework we used as an academic-public partnership to launch the VMP and how researchers can use grant funding as an advocacy mechanism to influence policy, ultimately supporting brain health and caregivers.

